# ATOX1 overexpression mitigates copper homeostasis in microglia: Implications for Alzheimer's disease therapy

**DOI:** 10.1016/j.gendis.2025.101888

**Published:** 2025-10-18

**Authors:** Fuxin Zhong, Jiani Wu, Zhangjing Deng, Wuhan Yu, Jiaqi Song, Yingxi Chen, Weihua Yu, Yang Lü

**Affiliations:** a Department of Geriatrics, The First Affiliated Hospital of Chongqing Medical University, Chongqing 400016, China; b Key Laboratory of Major Brain Disease and Aging Research (Ministry of Education), Chongqing Medical University, Chongqing 400014, China; c Institute for Brain Science and Disease, Chongqing Medical University, Chongqing 400016, China; d Institute of Neuroscience, Department of Human Anatomy, Chongqing Medical University, Chongqing 400016, China

**Keywords:** Alzheimer's disease, ATOX1, Copper, Microglia, Oxidative stress

## Abstract

Copper (Cu^2+^) is a known contributor to the pathogenesis of Alzheimer's disease (AD). However, it is uncertain whether proteins regulating copper homeostasis affect Cu^2+^ in microglia. Antioxidant protein 1 (ATOX1) plays a key role in Cu^2+^ homeostasis, oxidative stress, and cell protection. Despite its critical functions, the role of ATOX1 in AD pathology remains poorly defined. This study aims to examine the effects of ATOX1 on oxidative stress, apoptosis, and neuroinflammation in microglia by modulating Cu^2+^ homeostasis. *In vivo*, a 5 × FAD mouse model was used to investigate the localization and expression of ATOX1 in AD by immunofluorescence and three-dimensional reconstruction. The Aβ_1-42_ oligomer was used to establish an AD model *in vitro*. The role of ATOX1 in Cu^2+^ homeostasis regulation in microglia was further studied using co-immunoprecipitation, Western blotting, quantitative real-time PCR, and spectrophotometry. A reduction in ATOX1 expression was noted in Aβ-plaques-associated microglia compared with normal microglia. Cu^2+^ levels were elevated in the *in vitro* AD model, and ATOX1 directly regulated copper homeostasis via P1B-ATPase (ATP7B) in microglia. Excessive Cu^2+^ induced oxidative stress, neuroinflammation, and apoptosis. Overexpression of ATOX1 alleviated this neurotoxicity, indicating its potential to alleviate oxidative stress, cell apoptosis, and neuroinflammation in AD. ATOX1 overexpression offers protective effects on microglia through Cu^2+^ homeostasis, which may lead to potential therapeutic strategies for AD.

## Introduction

Alzheimer's disease (AD) is the most common form of dementia, characterized by a gradual decline in memory and cognitive functions.^1^ The hallmark of AD pathology is the presence of β-amyloid (Aβ) plaques.^2^ Numerous studies suggest that oxidative stress plays a key role in AD. Research has shown that copper (Cu^2+^) can aggregate and generate reactive oxygen species (ROS) in the brain.^3^ Cu^2+^ interacts with Aβ, facilitating various redox reactions.^4^ Recent studies have confirmed that the oxidative stress observed in AD is linked to the interaction between Cu^2+^ and Aβ, leading to ROS production.^5^ Moreover, several studies have indicated an increase in Cu^2+^ concentration near Aβ-plaques in both AD patients and mouse models.^6^, ^7^, ^8^ However, the precise mechanism of copper accumulation at the amyloid site remains unclear.

Heavy metals are stressors that induce the accumulation of ROS, apoptosis, and neuroinflammation.^9^^,^^10^ Numerous studies have shown that prolonged exposure to Cu^2+^ leads to ROS accumulation, resulting in mitochondrial dysfunction and apoptosis.^11^, ^12^, ^13^ Apoptosis serves as an intracellular defense mechanism, and the changes in Cu^2+^ levels can affect apoptosis signal transduction.^14^ Previous research highlighted the role of anti-apoptotic factors, such as the B-cell lymphoma 2 (Bcl2) family, in regulating the response of microglia to Aβ-plaques.^15^^,^^16^ Dysregulated Cu^2+^ levels can disrupt the inflammatory balance, as evidenced by the Cu^2+^ accumulation in microglia near Aβ-plaques and the resulting neurotoxic effects.^4^ Some studies suggest that chronic inflammatory responses in microglia near Aβ-plaques may be driven by Cu^2+^, potentially contributing to Aβ-plaque deposition and subsequent neuroinflammation.^17^ Overall, Cu^2+^ plays a significant role in oxidative stress, apoptosis, and neuroinflammation of AD cells. However, the specific mechanisms underlying its pathogenesis remain unclear.

Antioxidant protein 1 (ATOX1), initially discovered in yeast, protects cells from oxidative damage caused by superoxide and hydrogen peroxide.^18^ It plays a key role in intracellular copper distribution. In healthy cells, ATOX1 delivers Cu^2+^ to copper ATPases, including transmembrane P1B ATPase (ATP7B), located on the membrane of secretory vesicles and the trans Golgi apparatus network (TGN). As a copper chaperone, ATOX1 is essential for Cu^2+^ secretion, facilitating its efflux from the cell.^19^ Cells lacking ATOX1 exhibit compromised Cu^2+^ efflux, leading to copper accumulation and eventual cell death.^20^ Overexpression of ATOX1 enhances cellular antioxidant defenses in a copper-dependent manner.^18^ However, the roles of ATOX1 in AD are still unclear.

This study investigated the role of ATOX1 in mitigating oxidative stress by reducing copper levels in microglia through ATP7B. In 5 × FAD mice, decreased ATOX1 expression was observed in microglia associated with Aβ-plaques. In BV2 cells treated with Aβ_1-42_, ATOX1 interacted with ATP7B to facilitate the transfer of Cu^2+^ to ATP7B. Overexpression of ATOX1 attenuated Cu^2+^ accumulation and oxidative stress in microglia, ultimately decreasing cell apoptosis and neuroinflammation. This phenomenon may be due to a reduction in Cu^2+^ levels following ATOX1 overexpression in microglia adjacent to Aβ-plaques, potentially serving as a protective mechanism in AD.

## Materials and methods

### Animals

In this study, C57BL/6 mice were hybridized to create 5 × FAD mice as the AD model. Five AD-related mutations were observed in 5 × FAD mice, with the model exhibiting significant Aβ-plaque deposition and cognitive impairment at 12 weeks.^21^ Five mutations associated with AD have been identified in 5 × FAD mice, including the mutations in the human APP protein (Swedish type: K670N/M671L; Florida type: I716V; and London type: V717I) and mutations in the human PSEN1 protein (M146L and L286V).^22^ In the study, male 5 × FAD transgenic mice were bred with female C57BL/6J mice. The progeny was separated from their mother and transitioned to solid food at around four weeks old, and genotype was determined using PCR analysis.^23^ All 5 × FAD mice are transgenic heterozygotes. Throughout the experiment, due to known differences in disease progression between genders in the 5 × FAD mice, pathological differences related to amyloid plaques in the brain of 5-month-old male mice with 5 × FAD were evaluated, including cortical and hippocampus regions.^24^ The mice were collectively placed in our specific pathogen-free facility and maintained under 12-h light and 12-h dark conditions, with free access to food and water. All procedures for this study were approved by the Animal Care and Use Committee of Chongqing Medical University (ID: IACUC-CQMU-2024-0626).

### Reagents and antibodies

The following antibodies were used: cluster of differentiation 68 (CD68), ionized calcium binding adaptor molecule 1 (IBA1), neuronal nuclei (NeuN), and glial fibrillary acidic protein (GFAP) were from Bio-Rad, Novus, Pronteintech, and Abcam, respectively. Antibodies against ATOX1 for immunofluorescence analysis were obtained from Pronteintech (26641-1-AP). ATOX1 and ATP7B antibodies used for Western blotting and co-immunoprecipitation analysis were purchased from Santacruz (sc-100557, sc-373963). 1,1,1,3,3,3-hexafluoro-2-propanol (HFIP) was purchased from Sigma–Aldrich. Aβ_1–42_ was purchased from Nanjing TG peptide. As previously mentioned, Aβ_1–42_ was completely dissolved in HFIP using sonication. After dispensing the solution into appropriate volumes, the solvent was evaporated and the resultant Aβ_1–42_ was stored at −80 °C before use.^25^

### Lentivirus preparation

ATOX1 overexpression lentivirus was constructed by Beijing Tsing Biological Company. The lentivirus was used to transfect the BV2 cell lines. Subsequently, purithromycin was used for drug screening to construct an ATOX1-OE stable transgenic BV2 cell line.

### BV2 cell culture conditions

The BV-2 cell line has undergone thorough characterization and was obtained from Shanghai Zhongqiao Xinzhou Biotechnology Co., LTD. The mouse microglial BV2 cell line was grown and routinely maintained in high-glucose Dulbecco's Modified Eagle Medium (DMEM) (Gibco) supplemented with 10% fetal bovine serum (Gibco) and 1% penicillin/streptomycin (Gibco), and cultured in a humidified 5% CO_2_ atmosphere at 37 °C.

### Quantitative reverse transcription PCR

Total RNA of cultured cells and tissues was isolated using FastPure Cell/Tissue Total RNA Isolation Kit (Vazyme), and reverse-transcribed with the ABScript III RT Master Mix qPCR with gDNA Remover (ABclonal) according to the manufacturer's protocol. Real-time PCR was performed on the Biorad 96 Touch machine (Biorad) with 2 × Universal SYBR Green Fast qPCR Mix (ABclonal). β-actin was used as the internal control. The cycle threshold (Ct) of the gene transcript was standardized by the average Ct of housekeeping gene β-actin transcripts amplified in each reaction. The relative quantification of normalized transcript levels was calculated using the comparative Ct method (^ΔΔ^Ct). The sequences of PCR primers for the genes examined are listed below: Forward (ATOX1): 5′- GGGAGGAGTGGAGTTCAACATTG-3′; Reverse (ATOX1): 3′- TGCCTCTTCAGTTTATATCCGGAA-5′; Forward (β-actin): 5′- AGTGTGACGTTGACATCCGTA -3′; Reverse (β-actin): 3′- GCCAGAGCAGTAATCTCCTTC-5′.

### Western blot analysis

Cultured cells or tissues were lysed in lysis buffer (RIPA, Pronteitech) containing phosphatase inhibitor Single-Use Cocktail (#1861281, Pronteitech). Subsequently, a probe-tip sonicator set at level 2 for 10 s (Fisher Scientific 550 Sonic Dismembrator) was used on ice, followed by centrifugation at 12,000 r.p.m at 4 °C for 16 min. The proteins were separated by polyacrylamide gel electrophoresis and transferred onto polyvinylidene difluoride membranes. The membranes were blocked with 5% nonfat dry milk in Tris-buffered saline-Tween solution at room temperature for 1 h and then incubated with antibodies overnight at 4 °C. After washing, secondary antibodies conjugated with horseradish peroxidase (Biosharp) were applied. For quantification, band intensities were normalized to β-actin and averaged from at least three independent experiments.

### Immunostaining, imaging, and quantification

To facilitate immunofluorescence staining of brain tissue, mice were anesthetized and perfused with cold phosphate-buffered saline through the heart to extract the right hemisphere of the brain. Subsequently, the brain specimens were fixed at 4 °C in a 4% paraformaldehyde solution overnight. Following fixation, the brains were immersed in a 30% sucrose solution at 4 °C until they reached a state of sinking. Coronal brain slices measuring 40 μm in thickness were then obtained using a sliding microtome with a freezing stage. The brain sections were washed three times with phosphate-buffered saline, followed by permeabilization and blocking in blocking buffer (1% bovine serum albumin and 0.1% Triton X-100 in phosphate-buffered saline) at room temperature for 1 h. Subsequently, the sections were incubated with primary antibodies in blocking buffer at 4 °C overnight. Following a series of washes, the secondary antibodies were added to the blocking buffer for 1 h. Then, the samples were washed and re-stained with dihydrochloride for 20 min. An Olympus confocal microscope was used for image acquisition. Image analysis was conducted utilizing a custom macro programmed within ImageJ software to quantify the staining of ATOX1-positive microglia. A standard threshold was chosen and uniformly applied to all channels of each image before measurement. The quantification of ATOX1 immunofluorescence involved normalizing the total area of ATOX1 immunofluorescence to the total area of IBA1 immunofluorescence.

### Measurement of copper level in cells

As mentioned above, the copper levels in BV2 cells were detected using a commercial copper colorimetric analysis kit (E-BC-K300-M, Elabscience).^26^ The optical density was measured at the wavelength of 580 nm, and the copper concentration was calculated.

### Three-dimensional reconstruction

The 40 μm coronal slices were captured using an oil objective on a confocal microscope (Olympus). Imaging parameters were standardized for all experiments, and Z-stacking was conducted with 1.0 μm increments in the Z direction. Three-dimensional reconstruction of the selected images was carried out using Imaris 9.0.1 software (Bitplane).

### Detection of GSH, MDA, and SOD

Malondialdehyde (MDA), superoxide dismutase (SOD), and Glutathione (GSH) assay kits (BC0020, BC0170, BC1175, Solarbio) were used to detect intracellular MDA, SOD, and GSH levels according to the manufacturer's protocols. ImageJ software 1.26 was used for quantification.

### Identification of ATOX1 differentially expressed between the AD and normal samples

GSE33000, GSE48350, and GSE5281 microarray data were downloaded from the Gene Expression Omnibus (GEO) database (http://www.ncbi.nih.gov/geo). The raw data were downloaded as MINiML files. Box plots were drawn by GraphPad Prism 9.

### Statistical analysis and reproducibility

The statistical significance of the difference between the two groups was determined using the unpaired two-tailed Student's *t*-test or Mann–Whitney *U* test based on the normality test. The latest version of GraphPad Prism 9 (GraphPad Software) was used for statistics and graph images. No data were excluded from the analyses. All values were reported as mean ± standard error of the mean.

## Results

### ATOX1 decreased in AD

ATOX1, a key component of copper-transporting proteins, drew our attention. To explore its potential role in AD, we used the GEO database with “Alzheimer's disease” as the search term, selecting three large-scale datasets. First, we analyzed that the mRNA expression of ATOX1 decreased in an AD patient (Fig. S1A–S1C).

**Figure 1:**
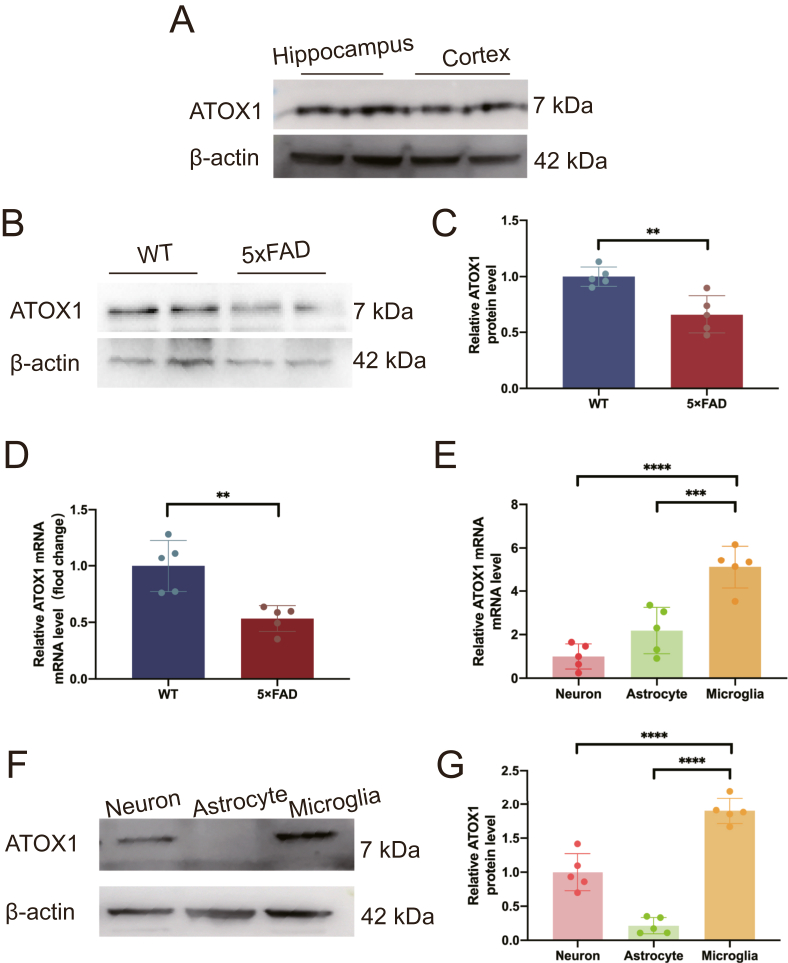
Expression of ATOX1 in Alzheimer's disease. **(A)** Western blot analysis of ATOX1 protein levels in the cortical and hippocampal samples from 5-month-old C57BJ/L mice. **(B)** Western blot analysis of ATOX1 protein levels in the hippocampal samples from 5-month-old wild-type and 5 × FAD mice. **(C)** Quantifications of ATOX1 protein levels. **(D)** The mRNA levels of ATOX1 in wild-type and 5 × FAD mice were detected by real-time PCR. ∗∗*p* < 0.01; *t*-test and Tukey's post hoc analysis. **(E)** Quantifications of ATOX1 mRNA levels in neurons, astrocytes, and microglia. **(F)** The ATOX1 protein levels in neurons, astrocytes, and microglia were detected by Western blot analysis. **(G)** Quantifications of ATOX1 protein levels. ∗∗∗*p* < 0.001, ∗∗∗∗*p* < 0.0001; data were reported as mean ± standard error of the mean; one-way ANOVA with Tukey's post hoc analysis.

Previous studies have documented the expression of ATOX1 in rodent brains, particularly in the hippocampus and cortex.^27^ The 5 × FAD transgenic mice employed in this study were generated on a C57BL/6 genetic background. Firstly, we confirmed ATOX1 presence in both the hippocampus and parietal cortex of C57BL/6 (Fig. 1A). The bilateral hippocampal tissues from 5-month-old 5 × FAD mice were then analyzed, revealing a significant down-regulation of ATOX1 expression compared with age-matched wild-type controls (*p* = 0.0015, *p* = 0.0046; Fig. 1B–D). To determine cell-type specificity of ATOX1 expression in the central nervous system, we quantified its levels in primary neurons, astrocytes, and microglia. Notably, ATOX1 was expressed in both neurons and microglia, with the highest abundance in microglia (*p* = 0.0002, *p* = 0.02; Fig. 1E–G). While prior reports confirmed ATOX1 presence in the central nervous system, its hippocampal distribution remained incompletely characterized.^28^^,^^29^

**Figure 2:**
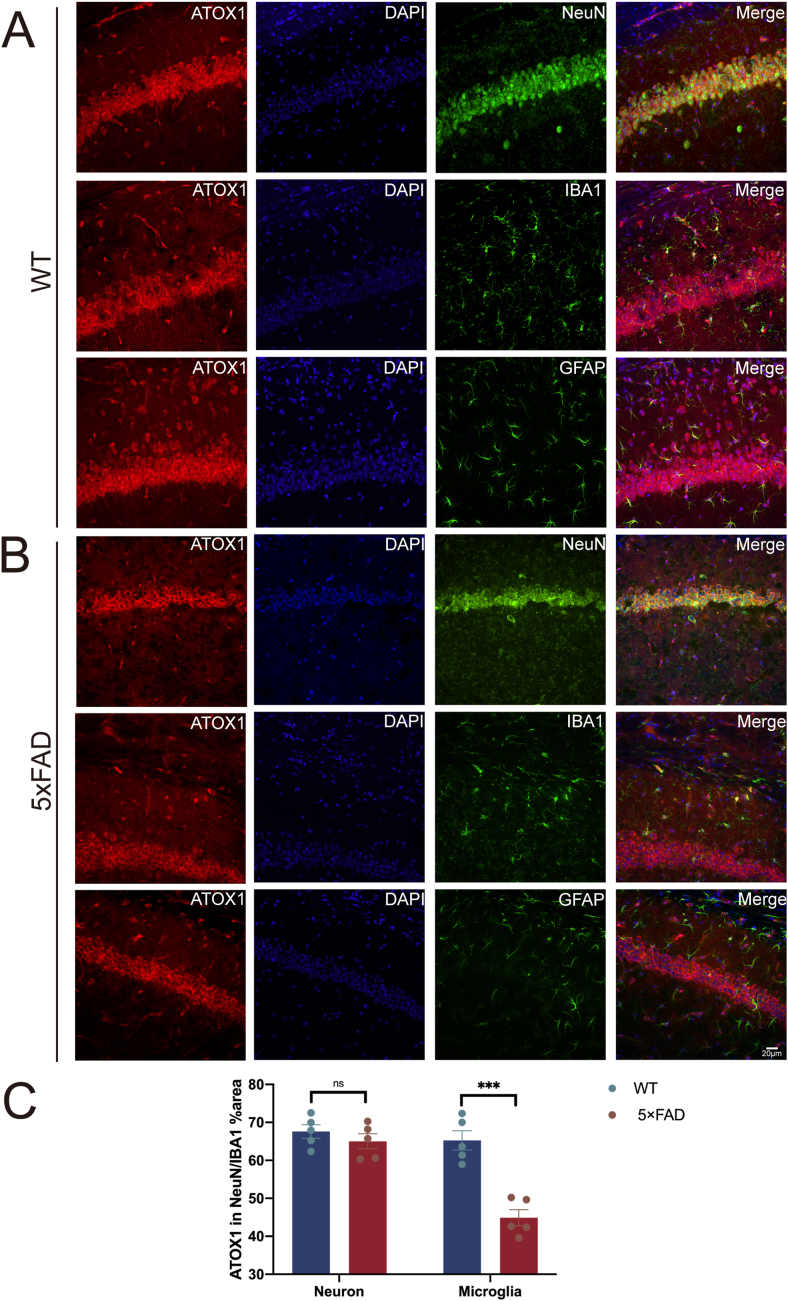
Cellular localization of ATOX1 in wild-type and AD mice. **(A)** Immunofluorescent staining of ATOX1 (red) and neuronal nuclei (NeuN)/ionized calcium binding adapter molecule 1 (IBA1)/glial fibrillary acidic protein (GFAP) (green) in the hippocampus from wild-type mice. **(B)** Immunofluorescent staining of ATOX1 (red) and NeuN, IBA1, and GFAP (green) in the hippocampus from 5-month-old male 5 × FAD mice. *n* = 5 in each group. Scale bar: 20 μm. **(C)** Quantification of ATOX1 intensity in neurons and microglia. ^ns^*P* > 0.05, ∗∗∗*p* < 0.001; data were reported as mean ± standard error of the mean; *t*-test and Tukey's post hoc analysis.

Immunofluorescence co-localization studies revealed ATOX1 expression in both neurons and microglia within the hippocampal region (Fig. 2A). However, ATOX1 fluorescence intensity was selectively diminished in microglia of 5 × FAD mice, while the expression in neurons remained unchanged (Fig. 2B and C; *p* = 0.0003 in microglia; *p* > 0.05 in neurons).

### ATOX1 was expressed in Aβ-plaque-associated microglia in AD

**Figure 3:**
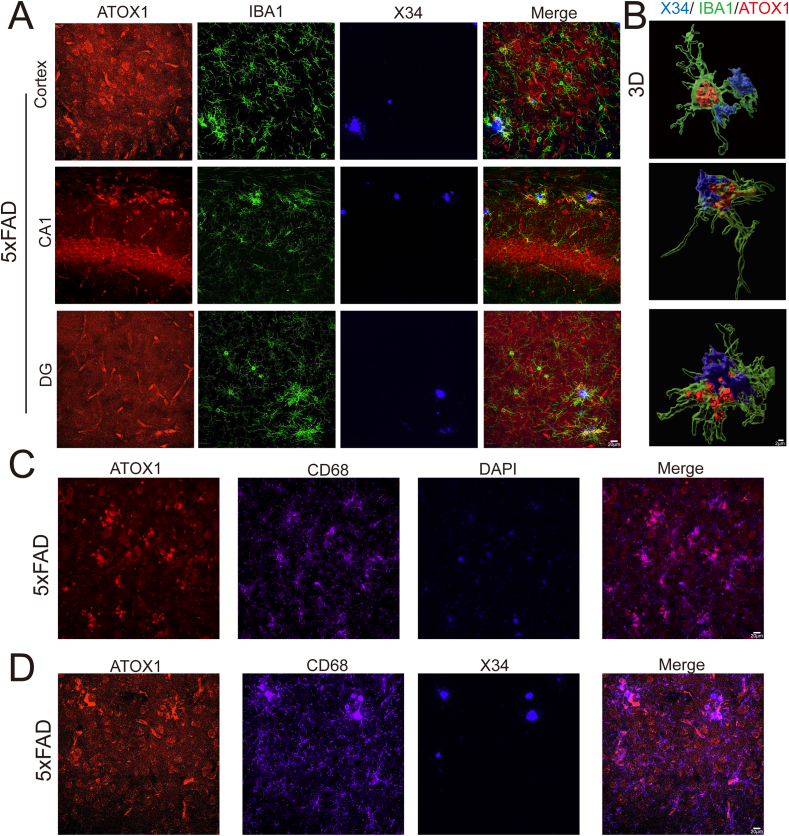
ATOX1 decreased in Aβ-plaque-associated microglia in AD. **(A)** Immunofluorescent staining of ATOX1 (red), X34 (blue), and IBA1 (green) in the hippocampus from 5-month-old 5 × FAD mice. Scale bar: 20 μm. **(B)** Representative three-dimensional images reconstruction after immunofluorescence triple labeling of ATOX1 (red), X34 (blue), and IBA1 (green) in hippocampal regions from 5-month-old 5 × FAD mice. Scale bar: 5 μm. **(C)** Double immunofluorescent staining of ATOX1 (red) and CD68 (magenta) from 5-month-old 5 × FAD mice. **(D)** Immunofluorescent staining of ΑTOX1 (red), X34 (blue), and CD68 (magenta) from 5-month-old 5 × FAD mice. Scale bar: 20 μm.

Microglia are typically in a resting state. Under pathological conditions, such as Aβ-plaques, microglia become activated and localize around to Aβ before phagocytosing them.^30^^,^^31^ These microglia are referred to as plaque-associated microglia. First, immunofluorescence was used to examine the association between ATOX1^+^ microglia and Aβ-plaques in the hippocampus and cortex of 5 × FAD mice. Notably, most ATOX1^+^ microglia were located around IBA1^+^ microglia surrounding Aβ-plaques (Fig. 3A). Three-dimensional reconstruction confirmed the co-localization of ATOX1^+^ microglia and Aβ-plaques (Fig. 3B). Additionally, we observed lower expression of ATOX1 in non-plaque-associated microglia (Fig. S2A and S2B; *p* = 0.0172).

In AD, CD68-labeled lysosomes are crucial for the phagocytosis of Aβ-plaques by microglia. CD68^+^ microglia are considered key for clearing Aβ.^32^, ^33^, ^34^ The results demonstrated a high degree of co-localization between the phagocytic marker CD68 and ATOX1 in 5 × FAD mice, suggesting that ATOX1-positive microglia may possess enhanced phagocytic activity (Fig. 3C). Further immunofluorescence analysis revealed significant overlap between CD68 and ATOX1 in IBA1^+^ microglia near Aβ-plaques (Fig. 3D), indicating that ATOX1 may influence microglial phagocytic function in AD pathology. The co-localization in AD suggests ATOX1 involvement in microglial activation. The observed Aβ-plaque-associated co-localization further implies the potential role of ATOX1 in regulating phagocytic function.

### ATOX1 reduced copper overload in BV2 cells through ATP7B

**Figure 4:**
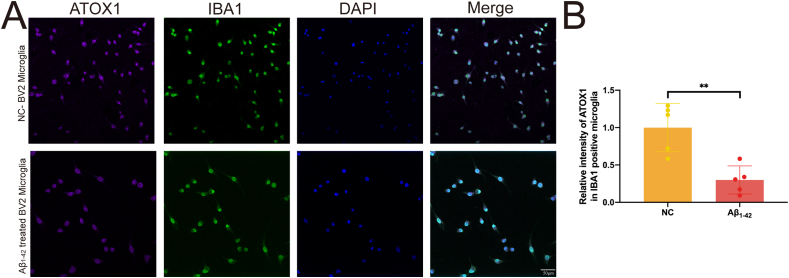
ATOX1 is reduced in Aβ_1-42_-treated BV2 cells. **(A)** Immunofluorescence analysis of ATOX1 (red) and IBA1 (green) in BV2 microglia with or without Aβ_1-42_. ∗∗*p* < 0.01; data were reported as mean ± standard error of the mean; *t*-test and Tukey's post hoc analysis.

ATOX1 is the primary copper chaperone in the cytoplasm. To investigate its role, we conducted semi-quantitative analysis using immunofluorescence. After treating BV2 cells with Aβ_1-42_, we observed a significant reduction in ATOX1 expression (Fig. 4A and B; *p* = 0.003).

**Figure 5:**
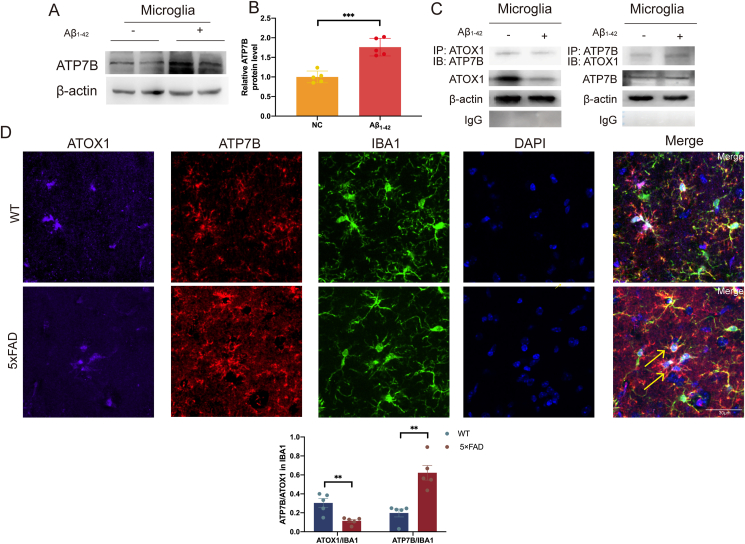
ATOX1 exerts a functional role in AD through interaction with ATP7B. **(A, B)** Expression of the main copper export protein ATP7B was detected in BV2 microglia with or without Aβ_1-42_ by Western blot analysis. ATP7B protein levels were quantified. **(C)** Detection of the interaction of ATOX1 and ATP7B on BV2 microglia cells treated with Aβ_1-42_ by co-immunoprecipitation. **(D)** Immunofluorescent staining of ATOX1 (magenta), ATP7B (red), IBA1 (green), and DAPI (blue) in the brain from 5-month-old wild-type and 5 × FAD mice. Scale bar: 30 μm. **(E)** Quantification of ATOX1 and ATP7B intensity in microglia. ∗∗*p* < 0.01, ∗∗∗*p* < 0.001; data were reported as mean ± standard error of the mean; *t*-test and Tukey's post hoc analysis.

Previous studies have shown that ATP7B plays a role in removing excess copper from the plasma membrane of cells.^35^, ^36^, ^37^ Based on these findings, we hypothesized that ATOX1 might function through ATP7B in BV2 cells. To test this hypothesis, we extracted cellular proteins after Aβ_1-42_ treatment and detected a significant increase in ATP7B expression in the Aβ_1-42_ group (Fig. 5A and B; *p* = 0.0002). To further explore the relationship between ATOX1 and ATP7B, co-immunoprecipitation confirmed their interaction (Fig. 5C). Additionally, immunofluorescence triple-labeling experiments performed on the brains of wild-type and 5 × FAD mice demonstrated clear co-localization of IBA1, ATOX1, and ATP7B in the hippocampus (Fig. 5D and E; *p* = 0.0053, *p* = 0.0013).

**Figure 6:**
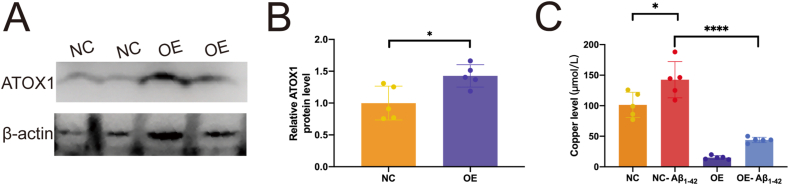
ATOX1 reduces copper overload in BV2 cells. **(A, B)** Lentivirus (LV) was used to mediate stable ATOX1 overexpression (OE). ATOX1 protein levels in BV2 cells with or without LV-OE were quantified. ∗*p* < 0.05; data were reported as mean ± standard error of the mean; *t*-test and Tukey's post hoc analysis. **(C)** The copper level in NC and OE in BV2 microglia with or without Aβ_1-42_. ∗*p* < 0.05, ∗∗∗∗*p* < 0.0001; data were reported as mean ± standard error of the mean; one-way ANOVA analysis followed by Tukey's multiple comparison test.

To explore whether ATOX1 activation affects copper accumulation via ATP7B, a lentivirus was used to overexpress ATOX1 in BV2 cells. Overexpression efficiency was found to be 63% (Fig. 6A and B; *p* = 0.0171). Further results revealed copper accumulation in BV2 cells following Aβ_1-42_ treatment (Fig. 6C; *p* = 0.0125). In contrast, overexpression of ATOX1 led to a significant reduction in intracellular copper concentrations (*p* < 0.0001; Fig. 6C). These findings suggest that ATOX1 regulates Cu^2+^ concentrations in microglia through its interaction with ATP7B, and that changes in ATOX1 expression are associated with Cu^2+^ accumulation in these cells.

### ATOX1 upregulation reduces oxidative stress, apoptosis, and neuroinflammation

**Figure 7:**
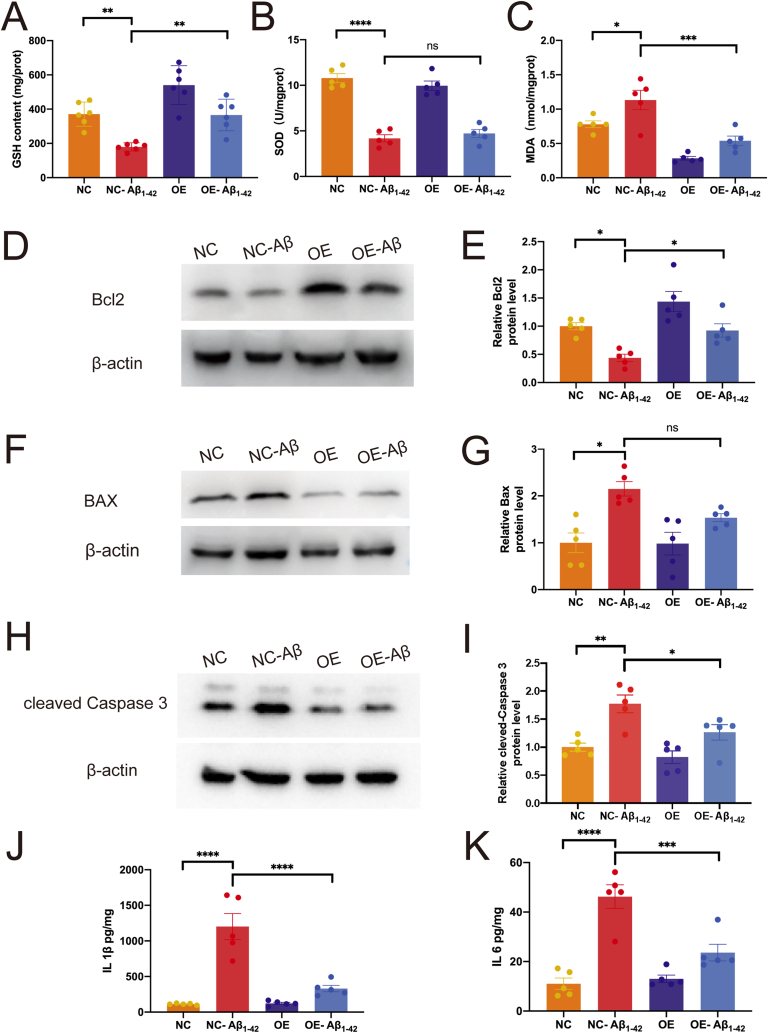
The up-regulation of ATOX1 attenuates oxidative stress, apoptosis, and neuroinflammation in BV2 cells. **(A**–**C)** Expression levels of antioxidants, superoxide dismutase (SOD), glutathione (GSH), and malondialdehyde (MDA), were measured in comparison to NC-BV2. **(D**–**I)** Western blot analysis was used to measure Bcl2, BAX, and cleaved caspase 3 expression in lentivirus-treated BV2 microglia with or without Aβ_1-42_. Quantifications of protein levels are shown in E, G, I. **(M, N)** The IL-1β and IL-6 levels in lentivirus-treated BV2 microglia with or without Aβ_1-42_ were measured by ELISA. ^ns^*P* > 0.05, ∗*p* < 0.05, ∗∗*p* < 0.01, ∗∗∗*p* < 0.001, ∗∗∗∗*p* < 0.0001; data were reported as mean ± standard error of the mean; one-way ANOVA analysis followed by Tukey's multiple comparison test.

The increase of Cu^2+^ in BV2 cells induced by Aβ_1-42_ requires many adaptive changes in the cells to prevent the toxicity from ROS. Next, the levels of antioxidants that protect cells from Cu^2+^ induced oxidative stress were tested. GSH has the capability to bind to a majority of cytoplasmic Cu^2+^, thereby promoting cell adaptation to elevated Cu^2+^ concentrations and mitigating resultant oxidative stress.^38^^,^^39^ The study observed a significant decrease in GSH expression in BV2 cells after Aβ_1-42_ treatment (*p* = 0.0033; Fig. 7A). After overexpression of ATOX1 and treatment with Aβ_1-42_, GSH increased compared with the NC-Aβ_1-42_ group (Fig. 7A; *p* = 0.0043). In the SOD activity assay, similar findings were observed in both the NC and NC-Aβ_1-42_ groups (Fig. 7B; *p* < 0.0001). Although no statistically significant difference was found between the OE-Aβ_1-42_ and NC-Aβ_1-42_ groups, an increase in SOD level was still observed (Fig. 7B; *p* > 0.05). It is also worth noting that the oxidative stress indicator MDA content was significantly reduced in the OE-Aβ_1-42_ group (Fig. 7C; *p* = 0.0006).

Previous studies have reported that excessive exposure to Cu^2+^ may lead to cell apoptosis.^11^ As shown in Figure 7D–I, compared with the NC group, Aβ_1-42_ significantly decreased the protein level of Bcl2 (Fig. 7D and E; *p* = 0.0402) and increased the protein level of Bax (Fig. 7F and G; *p* = 0.0105), and cleaved caspase 3 (Fig. 7H and I; *p* = 0.0088). In addition, compared with the NC-Aβ_1-42_ group, the protein levels of cleaved caspase 3 in BV2 microglia were significantly lower in the OE-Aβ_1-42_ group (Fig. 7H and I; *p* = 0.00391), but Bcl2 protein levels increased (Fig. 7D and E; *p* = 0.0055). Although the protein expression of Bax did not show statistical significance in the NC-Aβ_1-42_ and OE-Aβ_1-42_ groups, a decrease in Bax expression was observed in the OE-Aβ_1-42_ group (Fig. 7F and G; *p* > 0.05). In summary, these findings indicate that the level of Cu^2+^ increases after Aβ_1-42_ treatment, while excessive Cu^2+^ leads to cellular oxidative stress and induces BV2 apoptosis. Overexpression of ATOX1 can save this neurotoxicity. This indicates that ATOX1 can alleviate oxidative stress and cell apoptosis in microglia in AD.

The elevation of Cu^2+^ concentration results in a disruption of metal homeostasis, subsequently inducing the generation of ROS and perturbation of the inflammatory equilibrium.^40^ The synthesis of pro-inflammatory cytokines plays a role in neuroinflammation and contributes to the progression of AD.^41^ Our research centers on the multifunctional interleukin (IL)-1β and IL-6, which have been previously linked to inflammatory reactions in microglia in AD.^42^ Using an ELISA on BV2 cells, compared with the NC group, in the NC-Aβ_1-42_ group, the levels of IL-1β and IL-6 significantly increased (Fig. 7J and K; *p* < 0.0001). The levels of cytokines IL-1β and IL-6 significantly decreased in the AD model with ATOX1 overexpression (OE-Aβ_1-42_ group) (Fig. 7J and K; *p* < 0.0001). These results indicate that overexpression of ATOX1 can inhibit pro-inflammatory responses.

## Discussion

This study demonstrates that ATOX1 expression is markedly reduced in microglia in 5 × FAD cells. Notably, ATOX1 expression is reduced in Aβ-plaque-associated microglia compared with microglia. ATOX1 plays a critical role in maintaining Cu^2+^ homeostasis via ATP7B. Modulating ATOX1 expression alleviates oxidative stress, inhibits apoptosis, and reduces neuroinflammation in microglia. The relationship between microglial cell death and the improvement of AD pathology is complex. Microglia play multiple roles in AD, including neuron protection and clearance of pathological substances. However, excessive activation can trigger inflammation and exacerbate neuronal damage. In this study, we found that the compensatory addition of ATOX1 may have protective effects. It alleviates oxidative stress while reducing the negative impact of apoptosis and inflammation. This balance may help improve AD pathology. To our knowledge, this is the first study to demonstrate that ATOX1 provides neuroprotection in AD by preserving copper homeostasis in microglia.

Previous studies have shown that high concentrations of Cu^2+^ accumulate near Aβ-plaques in AD brain 43. In fact, some data from the AD mouse model indicate that Cu^2+^ chelation reduces Aβ-plaque deposition and improves cognitive function.^44^ ATOX1, a key cytoplasmic copper transporter, plays a crucial role in Cu^2+^ homeostasis. A study has found that ATPase is highly expressed in Cu^2+^-overloaded microglia and mediates Cu^2+^ transport.^45^ In cells, Cu^2+^ is transferred to ATP7B, located in the trans-Golgi network and inner vesicles, via ATOX1, and subsequently exported to the extracellular space by ATP7B. ATOX1 is essential for regulating cellular Cu^2+^ levels through this pathway.^45^^,^^46^ In this study, ATOX1 and ATP7B interacted in BV2 cells treated with Aβ_1-42_. Furthermore, ATP7B expression was up-regulated, while ATOX1 expression was down-regulated. These changes may contribute to the increased Cu^2+^ level in microglia. After ATOX1 loss, Cu^2+^ accumulation in the cytoplasm was directly observed.^47^ Our research showed that ATOX1 up-regulation reduced Cu^2+^ overload in microglia. The results suggest that the regulation of Cu^2+^ transport by ATOX1 and ATP7B may help maintain Cu^2+^ homeostasis in activated microglia at Aβ-plaque sites.

The bonding of Cu^2+^ to Aβ forms a stable complex, leading to increased ROS production in mouse brain tissue. High concentrations of Cu^2+^ can disrupt the oxidation/antioxidation balance in the brain tissue of AD patients, leading to ROS accumulation and oxidative stress damage.^47^ Cells use a variety of antioxidant systems to mitigate oxidative stress, including free radical scavenging enzymes and non-enzymatic antioxidants. SOD is considered the most important enzyme in combating ROS, as it catalyzes the conversion of superoxide into water and oxygen. MDA, a byproduct of lipid oxidation, exacerbates cellular membrane damage, making it a key indicator of membrane integrity. GSH, a low-molecular-weight scavenger, binds to most cytoplasmic Cu^2+^, enabling cells to tolerate high Cu^2+^ levels while mitigating oxidative damage.^38^ These molecules classically participate in the oxidation/antioxidant balance. Essential antioxidants are crucial in eliminating free radicals and maintaining redox equilibrium. Studies have shown that overexpression of ATOX1 can reduce MDA levels and increase GSH levels in BV2 cells stimulated by Aβ_1-42_, thereby enhancing their antioxidant capacity. This suggests that ATOX1 up-regulation may protect cells from oxidative stress by enhancing GSH levels. Previous research has shown that ATOX1 up-regulation can alleviate Cu^2+^-induced oxidative stress in neurons by reducing ROS levels.^48^ However, the exact protective mechanism of ATOX1 in AD remains unclear.

Oxidative stress is a key factor in inducing programmed cell death, specifically apoptosis, and neuroinflammation in microglia.^49^^,^^50^ Numerous studies have demonstrated a strong association between excessive microglia activation and oxidative stress.^51^, ^52^, ^53^ This hyperactivation not only facilitates the release of pro-inflammatory cytokines, but also triggers microglial apoptosis and dysfunction, thereby impairing the innate immune functions of the brain 54,55. It is suggested that oxidative stress may act as an indirect contributor to Cu^2+^ overload. The Bcl2 protein family plays a central role in regulating anti-apoptotic processes. Upon receiving apoptotic signals, Bcl2 family proteins will transfer from the cytoplasm to the mitochondrial membrane, where they exert anti-apoptotic effects. The ratio of Bcl2 to Bax is critical in determining whether a cell survives or undergoes apoptosis.^56^ Additionally, mitochondrial oxidative phosphorylation can release apoptosis-inducing factors into the cytoplasm, activating caspase-3 and further promoting apoptosis.^57^ Our findings showed that *in vitro*, Bcl2 expression was significantly decreased while pro-apoptotic proteins, including Bax and cleaved-caspase 3, were markedly increased, indicating a high rate of apoptosis. Conversely, overexpression of ATOX1 reversed these changes, suggesting a potential protective mechanism against apoptosis and oxidative damage. These findings support the potential of exogenous ATOX1 to reduce apoptosis in AD. In summary, our findings offer initial support for the potential protective role of ATOX1 in modulating microglial function in the context of AD. A previous study has suggested a link between microglial resistance to apoptosis and chronic inflammation.^58^ Furthermore, microglia can promote apoptosis through the release of various pro-inflammatory factors.^59^ The results show that the up-regulation of ATOX1 inhibits the expression of IL-1β and IL-6, thereby reducing inflammation. Those findings suggest that ATOX1 may help mitigate neuroinflammation in AD models. Consequently, investigating the role of ATOX1 in controlling AD has sparked interest due to its potential therapeutic implications.

Preliminary results indicate that ATOX1 is localized in both microglia and neurons. Since ATOX1 is predominantly expressed in microglia, subsequent *in vitro* experiments focused on its effects in these cells. Clarifying the role of ATOX1 in microglial function is essential in the context of AD. This study is the first to identify a potential association between ATOX1 and Aβ-plaque-associated microglia, possibly linked to Cu^2+^ regulation and antioxidant activity. Additionally, up-regulation of ATOX1 effectively protects microglia from oxidative stress damage, consequently reducing the incidence of apoptosis and neuroinflammation. A previous study has demonstrated that elevated Cu^2+^ levels can intensify oxidative stress-induced microglial apoptosis in an AD mouse model.^60^ This current finding suggests that ATOX1 may lower intracellular Cu^2+^ levels by modulating microglial activity. However, further studies are needed to clarify the mechanisms through which ATOX1 influences microglial function and contributes to AD pathogenesis.

## Conclusion

ATOX1 serves as a key link between Cu^2+^ and microglia in AD. The up-regulation of ATOX1 demonstrates a protective influence on microglia in AD, suggesting potential therapeutic avenues for addressing the pathogenesis of the condition.

## CRediT authorship contribution statement

**Fuxin Zhong:** Writing – original draft, Resources, Conceptualization. **Jiani Wu:** Supervision, Conceptualization. **Zhangjing Deng:** Methodology. **Wuhan Yu:** Formal analysis. **Jiaqi Song:** Supervision. **Yingxi Chen:** Visualization. **Weihua Yu:** Visualization, Supervision. **Yang Lü:** Writing – review & editing, Visualization.

## Ethics declaration

The study protocol was approved by the Ethics Committee of Chongqing Medical University. All animal studies were conducted in accordance with the principles outlined in the Animal Research: Reporting In Vivo Experiments guidelines.

## Data availability

The datasets used and/or analyzed during the current study are available from the corresponding author upon reasonable request. The raw data supporting the conclusions of this article will be made available by the authors without undue reservation.

## Funding

This study was supported by grants from the Chongqing Talent Plan (China) (No. cstc2022ycjh-bgzxm0184), the Key Project of Science and Technology Research Program of Chongqing Municipal Education Commission (China) (No. KJZD-K202200405), the Innovation Project for Doctoral Students at The First Affiliated Hospital of Chongqing Medical University (China) (No. CYYY-BSYJSCXXM-202320), and the Chongqing Medical Key Discipline and Regional Medical Key Discipline Development Project (China) (0201[2023] No. 160 202412).

## Conflict of interests

The authors declared no competing interests.
