# Supplementary material for: ATOX1 overexpression mitigates copper homeostasis in microglia: Implications for Alzheimer's disease therapy
Source: Genes Dis. 2025 Oct 18;13(4):101888. doi: 10.1016/j.gendis.2025.101888 (PMC13092486; doi:10.1016/j.gendis.2025.101888)
Supplement: Multimedia component 1 [file mmc1.docx]

**Supplement Figure 1.** A. Expression levels of ATOX1 in the human brains. Expression values are from brain samples with Normal and AD in the dataset GSE33000, GSE48350, and GSE5281, Pearson's r correlation coefficient was used for statistical testing.


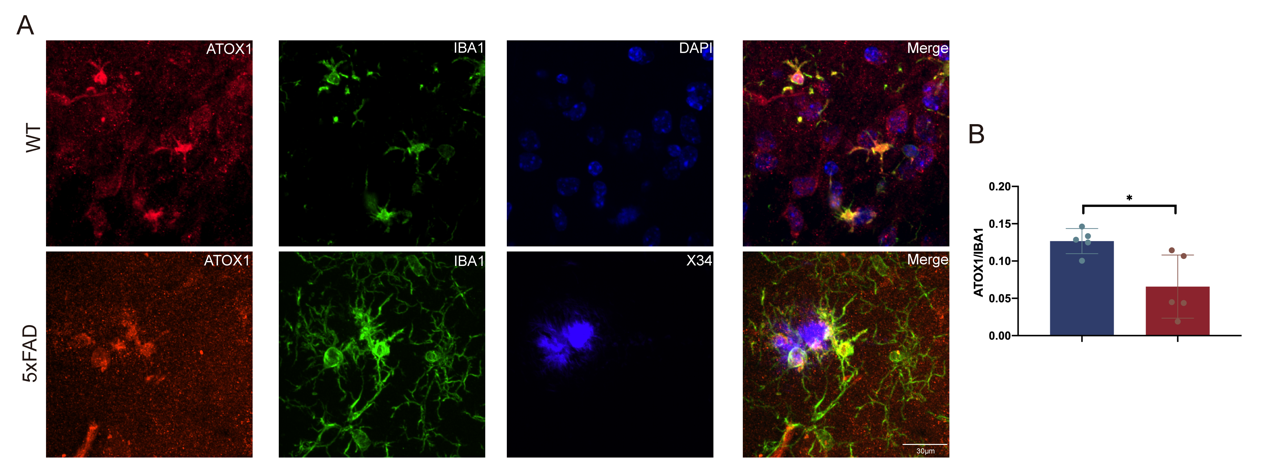


**Supplement Figure 2.** A. Immunofluorescent staining of ATOX1 (red), IBA1 (green) and DAPI/X34 (blue) in the brain from WT and 5×FAD mice. Scale bar: 30μm. B. Quantifications of ATOX1 intensity in microglia.
